# Left bundle branch area pacing in patients with heart failure and right bundle branch block: Results from International LBBAP Collaborative-Study Group

**DOI:** 10.1016/j.hroo.2022.05.004

**Published:** 2022-05-14

**Authors:** Pugazhendhi Vijayaraman, Oscar Cano, Shunmuga Sundaram Ponnusamy, Manuel Molina-Lerma, Joseph Y.S. Chan, Santosh K. Padala, Parikshit S. Sharma, Zachary I. Whinnett, Bengt Herweg, Gaurav A. Upadhyay, Faiz A. Subzposh, Neil R. Patel, Dominik A. Beer, Agnieszka Bednarek, Grzegorz Kielbasa, Roderick Tung, Kenneth A. Ellenbogen, Marek Jastrzebski

**Affiliations:** ∗ Geisinger Heart Institute, Wilkes-Barre, Pennsylvania; † Hospital Universitari i Politècnic La Fe, Valencia, Spain, and Centro de Investigaciones Biomédicas en RED en Enfermedades Cardiovasculares (CIBERCV), Spain; ‡ Velammal Medical College, Madurai, India; § Virgen de las Nieves Hospital, Granada, Spain; ‖ Chinese University of Hong Kong, Hong Kong; ¶ Virginia Commonwealth University Health System, Richmond, Virginia; # Rush University Medical Center, Chicago, Ilinois; ∗∗ Imperial College London, London, United Kingdom; †† University of South Florida, Tampa, Florida; ‡‡ University of Chicago, Chicago, Illinois; §§ The Wright Center, Scranton, Pennsylvania; ‖‖ Johns Hopkins University, Baltimore, Maryland; ¶¶ First Department of Cardiology, Interventional Electrocardiology and Hypertension, Jagiellonian University, Medical College, Krakow, Poland; ## University of Arizona, Phoenix, Arizona

**Keywords:** Cardiac resynchronization therapy, Left bundle branch area pacing, Right bundle branch block, Heart failure, Cardiomyopathy

## Abstract

**Background:**

Cardiac resynchronization therapy (CRT) using biventricular pacing has limited efficacy in patients with heart failure (HF) and right bundle branch block (RBBB). Left bundle branch area pacing (LBBAP) is a novel physiologic pacing option.

**Objective:**

The aim of the study was to assess the feasibility and outcomes of LBBAP in HF patients with RBBB and reduced left ventricular systolic function, and indication for CRT or ventricular pacing.

**Methods:**

LBBAP was attempted in patients with left ventricular ejection fraction (LVEF) <50%, RBBB, HF, and indications for CRT or ventricular pacing. Procedural, pacing, and electrocardiographic parameters; clinical response (no HF hospitalization and improvement in NYHA class); and echocardiographic response (≥5% increase in ejection fraction) to LBBAP were assessed.

**Results:**

LBBAP was attempted in 121 patients and successful in 107 (88%). Patient characteristics included age 74 ± 12 years, female 25%, ischemic cardiomyopathy 49%, and ejection fraction 35% ± 9%. QRS axis at baseline was normal in 24%, left axis 63%, right axis 13%. LBBAP threshold and R-wave amplitudes were 0.8 ± 0.3 V @ 0.5 ms and 10 ± 9 mV at implant and remained stable during mean follow-up of 13 ± 8 months. LBBAP resulted in narrowing of QRS duration (156 ± 20 ms to 150 ± 24 ms (*P* = .01) with R-wave peak times in V_6_ of 85 ± 16 ms. LVEF improved from 35% ± 9% to 43% ± 12% (*P* < .01). Clinical and echocardiographic response was observed in 60% and 61% of patients, respectively. Female sex and reduction in QRS duration with LBBAP were predictive of echocardiographic response and super-response.

**Conclusion:**

LBBAP is a feasible alternative to deliver CRT or physiologic ventricular pacing in patients with RBBB, HF, and LV dysfunction.

## Graphical abstract

**Figure:**
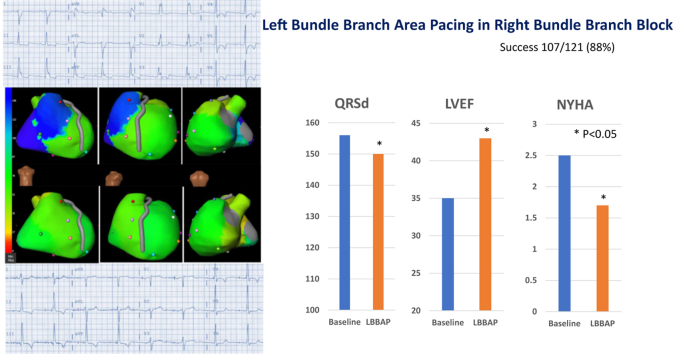

**Key Findings**

▪ Left bundle branch area pacing (LBBAP) was feasible in most patients with right bundle branch block (RBBB) requiring cardiac resynchronization therapy (CRT).
▪ LBBAP was attempted in 121 patients with RBBB eligible for CRT and was successful in 107 patients (88%).
▪ LBBAP was associated with improvement in clinical and echocardiographic outcomes in patients with RBBB and left ventricular dysfunction.
▪ Female sex and reduction in QRS duration with LBBAP were predictive of echocardiographic response and super-response.

## Introduction

**Figure 1:**
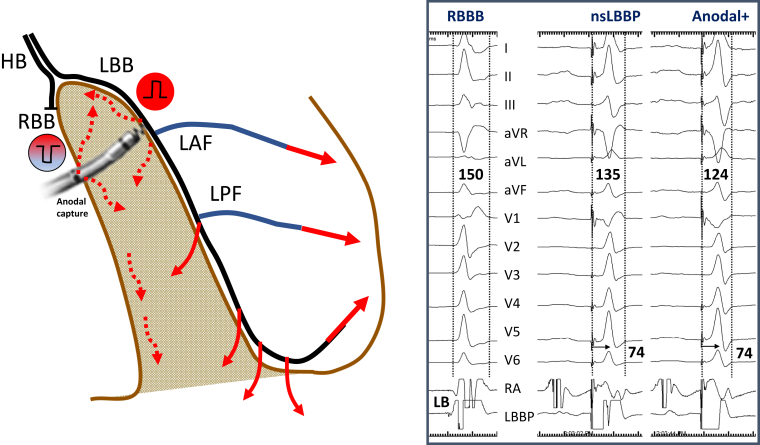
Schematic representation of left bundle branch pacing (LBBP) with anodal capture. Nonselective LBBP (nsLBBP) results in narrowing of native right bundle branch block (RBBB) from early left ventricular septal activation and anodal capture with simultaneous right ventricular septal activation. HB = His bundle; LAF = left anterior fascicle; LB = left bundle; LBB = left bundle branch; LPF = left posterior fascicle; RA =right atrium; RBB = right bundle branch.

**Figure 2:**
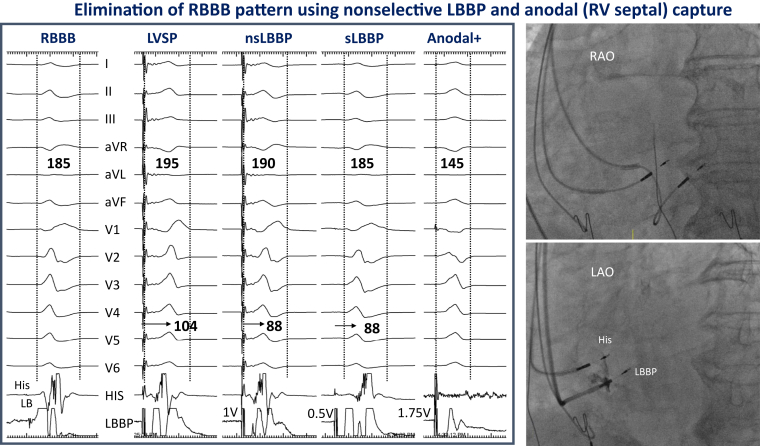
Left bundle branch pacing (LBBP) in a patient with right bundle branch block (RBBB). Twelve-lead electrocardiogram along with intracardiac electrograms from His and LBBP leads are shown at a sweep speed of 100 mm/s during left ventricular septal pacing (LVSP), nonselective (nsLBBP) and selective LBBP (sLBBP), and bipolar pacing with anodal (right ventricular [RV] septal) capture. Note the stimulus to peak R-wave times of 104, 88, and 88 ms during LVSP, nsLBBP, and sLBBP, respectively, with significant QRS reduction during anodal capture. Fluoroscopic views of LBBP and His leads are shown in right anterior oblique (RAO) and left anterior oblique (LAO) projections.

Cardiac resynchronization therapy (CRT) using biventricular pacing (BVP) is an effective therapy for patients with cardiomyopathy, reduced left ventricular ejection fraction (LVEF), heart failure (HF), and prolonged QRS duration. Multiple randomized trials have demonstrated improvements in quality of life/exercise capacity and reduction in HF-related hospitalization (HFH) and mortality, especially in patients with left bundle branch block (LBBB).^1^, ^2^, ^3^, ^4^, ^5^ Randomized clinical trials of BVP in patients with right bundle branch block (RBBB) compared to no pacing have not been performed. However, meta-analysis and systematic review of data from large clinical trials do not suggest favorable outcomes in patients with RBBB.^6^, ^7^, ^8^ Current guidelines provide BVP, a class IIa recommendation for patients with RBBB and QRS duration ≥150 ms and a class IIb recommendation for patients with RBBB and QRS duration of 120–150 ms.^9^ Recently, permanent His bundle pacing (HBP) was shown to achieve electrical resynchronization and improve clinical outcomes in patients with RBBB and reduced LVEF in a multicenter, observational study.^10^ However, HBP can be technically challenging and may be associated with higher pacing thresholds to correct RBBB and lower success rates, in addition to increased incidence of lead revisions.^11^^,^^12^ Over the last few years, left bundle branch area pacing (LBBAP) has emerged as an alternative form of physiologic pacing.^13^, ^14^, ^15^ LBBAP is technically easier to perform and is associated with low and stable capture thresholds and shown to improve clinical outcomes in patients requiring CRT.^16^, ^17^, ^18^ While LBBAP is often associated with right ventricular (RV) conduction delay, this may be physiologically quite different than RBBB owing to isolated conduction block as a result of concomitant capture of the interventricular septum and the left bundle branch (nonselective LBBAP) (Figure 1). Anodal capture of the RV septum may attenuate or eliminate the RBBB pattern in many patients (Figure 2). The aim of this multicenter study was to assess the safety, feasibility, and efficacy of LBBAP in patients with RBBB, HF, reduced LVEF, and indication for CRT or pacing.

## Methods

### Patient selection

This was a retrospective, multicenter, observational cohort study designed to evaluate the real-world experience of LBBAP in patients with RBBB, HF, and left ventricular (LV) dysfunction. The study population included patients with RBBB, QRS duration >120 ms, New York Heart Association (NYHA) class II–IV HF symptoms, baseline LVEF ≤50%, and indication for CRT or pacing. This international LBBAP collaborative study was performed at 11 centers (United States 5, Spain 2, India 1, Hong Kong 1, United Kingdom 1, and Poland 1). Patients provided informed consent and demonstrated an understanding of LBBAP as a nonstandard approach to achieve cardiac resynchronization / ventricular pacing. Data analysis was approved by the institutional review board at each site. The research reported in this paper adhered to Helsinki Declaration (as revised in 2013) guidelines. The data underlying this article will be shared on reasonable request to the corresponding author.

### Procedural details

In centers with experience, HBP was attempted first and if satisfactory electrical outcomes (acceptable His capture or bundle branch block correction thresholds) were not achieved, then LBBAP was attempted. In other centers LBBAP was chosen as the first-line therapy without attempting HBP. LBBAP was also attempted when coronary sinus (CS) lead placement was unsuccessful. LBBAP was performed using the SelectSecure^TM^ pacing lead (model 3830, 69 cm; Medtronic Inc, Minneapolis, MN), as previously described.^13^, ^14^, ^15^ Presence of Purkinje potentials recorded from the LBBAP lead and the potential to QRS onset intervals (LBB-V) were documented. Pacing thresholds were assessed by evaluating the transition from nonselective to selective left bundle branch (LBB) capture or nonselective LBB capture to LV septal myocardial capture. QRS narrowing during anodal capture, elimination or attenuation of RBBB pattern, baseline QRS axis, and changes in QRS axis were documented. Left axis deviation was defined as QRS axis of -30 to -90 degrees while right axis deviation was 90 to 180 degrees.

### Determination of left bundle branch capture

**Figure 3:**
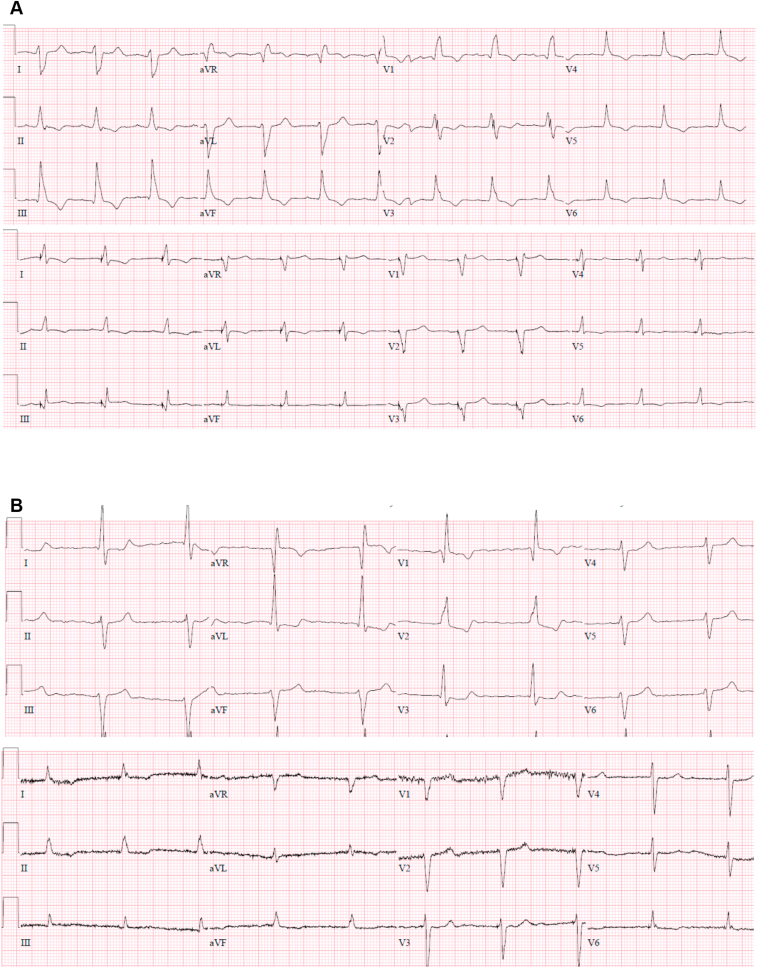
Correction of right bundle branch block (RBBB) with axis deviation. **A:** Left bundle branch area pacing (LBBAP) lead in the anterior septum in a patient with RBBB and right axis deviation with normalization of QRS axis and significant attenuation of RBBB pattern. **B:** LBBAP lead in the mid septum with correction of RBBB and left axis deviation.

During unipolar-tip pacing, right bundle branch morphology was observed in addition to 1 or more of the following findings: (1) LBB potentials (LBB-V intervals); (2) transition from nonselective to selective LBB capture or left septal myocardial capture at near-threshold outputs (Figure 2); (3) short and constant V_6_ peak LV activation times of <90 ms; (4) programmed stimulation (extrastimulus testing) to differentiate LV septal vs nonselective LBB capture. If LBB capture could not be confirmed, only LV septal capture was considered to be present.^14^, ^15^, ^16^, ^17^, ^18^, ^19^, ^20^

### Follow-up

Patients were followed in the device clinic at 2 weeks, 3 months, and 1 year and by remote monitoring every 3 months, when feasible. R-wave amplitudes, capture thresholds, lead impedance, and percentage of ventricular pacing were recorded at each visit. All capture thresholds were defined using a pulse width of 0.5 ms. QRS duration during pacing was measured from stimulus to the end of QRS. RBBB elimination was defined as no R′ in V_1_ with minimal or no S waves in V_5,6_. RBBB attenuation was decrease in the amplitude and width of R′ in V_1_ and S in V_5,6_. Lead-related complications were routinely tracked. The ejection fraction and LV volumes were calculated using Simpson’s biplane method. Echocardiographic indices, including LVEF, LV end-diastolic dimensions, and LV volumes, were recorded preimplant and at 3–6 months follow-up. Echocardiographic response was defined as a ≥5% increase in LVEF. Super-response was defined as an absolute improvement in LVEF by ≥20% or improvement of LVEF to >50% (in patients with LVEF ≤35%) between baseline and follow-up echocardiograms.^21^ Change in NYHA functional class, any HFH, and death from any cause were recorded.

Clinical response to CRT was defined as an improvement in NYHA functional class by at least 1 class and no HFH. HFH was defined as a hospital admission or an urgent care visit for intensive treatment for HF with intravenous diuretics or intravenous inotropic medications.

### Statistical analysis

Data were summarized using frequencies and percentages for categorical data and mean ± standard deviation or median and interquartile range for continuous variables. The descriptive statistics were reported for the full sample and stratified by success or failure to achieve LBBAP. Comparison between groups was accomplished using the χ^2^ or Fisher exact tests, and 2-sample *t* test or Wilcoxon rank sum test. Comparisons of continuous variables within groups were carried out with paired *t* test or Wilcoxon signed rank test. Univariate logistic regression analyses were used to estimate the odds ratios for achieving echocardiographic and clinical response as defined above for various baseline characteristics. Multivariate regression analysis was then performed on variables with odds ratios with *P* < .10. A backwards stepwise regression method was then used to determine the final multivariate regression model. Statistical analysis was performed using SPSS (version 27; SPSS Inc, Chicago, IL). A *P* value of less than .05 was considered significant.

## Results

A total of 121 patients underwent an attempt at LBBAP at 11 centers. Patients were followed for an average duration of 13 ± 8 months (median 9 months; range 0–53 months). Two patients were lost to follow-up.

### Baseline characteristics

Baseline characteristics of the study population are shown in Table 1. Mean age of the patients was 74 ± 12 years and 25% were female. All patients had preexisting RBBB and cardiomyopathy at baseline with mean LVEF of 35% ± 9% (57% of patients had LVEF ≤35% and 43% had LVEF between 35% and 50%). Fifty-three percent of patients had NYHA functional class III–IV. Forty-nine percent of patients had ischemic cardiomyopathy. Baseline QRSd of the entire cohort was 157 ± 20 ms, while 63% of patients had QRSd >150 ms. Normal QRS axis was observed in 24% while right or leftward axis was present in 14% and 62% of patients, respectively.

**Table 1:** Baseline characteristics

Characteristic	All patients (N = 121)	Successful (N = 107)	Failed (N = 14)	*P* value
Age, y	74 ± 12	74 ± 12	75 ± 8	.82
Female	30 (25)	26 (24)	4 (29)	.73
Medical history				
HTN	91 (75)	80 (75)	11 (79)	.76
DM	44 (36)	40 (37)	4 (29)	.52
CAD	69 (57)	59 (55)	10 (71)	.25
AF	50 (41)	44 (41)	6 (43)	.75
Ischemic cardiomyopathy	59 (49)	49 (46)	10 (71)	.08
Baseline NYHA class 3 or 4	64 (53)	56 (52)	8 (57)	.42
Baseline NYHA	2.5 ± 0.9	2.5 ± 0.8	2.7 ± 0.8	.54
Echocardiographic parameters				
LVEF	35 ± 9	35 ± 9	34 ± 9	.55
LVEDD (mm)	55 ± 12	54 ± 12	61 ± 8	.05
LVESV (mL)	96 ± 49	95 ± 46	99 ± 52	.06
LVEDV (mL)	138 ± 62	136 ± 61	142 ± 54	.10
LA volume index (mL/m^2^)	55 ± 31	54 ± 30	58 ± 33	.04
Electrocardiographic parameters:				
Baseline QRS (ms)	157 ± 20	156 ± 20	172±16	<.01
Baseline QRS >150 ms	76 (63)	62 (58)	14 (100)	<.01

Values are mean ± SD or n (%).

AF = atrial fibrillation; CAD = coronary artery disease; DM = diabetes mellitus; HTN = hypertension; LA = left atrial; LVEDD = left ventricular end-diastolic diameter; LVEDV = left ventricular end-diastolic volume; LVEF = left ventricular ejection fraction; LVESV = left ventricular end-systolic volume; NYHA = New York Heart Association.

### Feasibility

LBBAP was successful in 107 of 121 patients (88%). LBBAP was unsuccessful in 14 patients; deep septal penetration was not feasible in 6 patients and no significant narrowing of QRS was noted in the remaining 8 patients in whom BVP with CS lead was subsequently performed. Patients who failed deep septal lead implantation had wider QRS at baseline (172 ± 16 ms vs 156 ± 20 ms; *P* < .01) and larger left atrial volumes (54 ± 30 mL/m^2^ vs 58 ± 33 mL/m^2^; *P* = .04).

HBP was initially attempted in 55 patients: high threshold (>2.5 V @ 1 ms) to correct RBBB was observed in 27 patients; no correction was achieved in 12 patients; and His bundle could not be mapped in 16 patients. In the remaining patients, HBP was not attempted. In 5 patients, LBBAP was successfully performed as a rescue strategy after a failed attempt at CS lead implant.

Procedural outcomes are presented in Table 2. Forty-three percent of patients received a CRT device (implantable cardioverter-defibrillator [ICD] 32% and pacemaker 11%), while the remainder (57%) received a dual-chamber or single-chamber device (pacemaker 54%, ICD 3%). In patients receiving a CRT device, the LBBAP lead was always connected to the LV port. In most patients LV-RV delay was programmed to 80 ms to avoid RV pacing. In 12% of patients, fusion with pacing from the RV lead was used to achieve QRS narrowing. Anodal capture (bipolar) from the LBBAP lead (<3 V) was used in 48% of patients to achieve QRS narrowing. In patients with chronic atrial fibrillation and ICD indication, the LBBAP lead was connected to the atrial port and the device programmed to DDIR mode. Total procedural duration was 97 ± 48 minutes while the fluoroscopy duration was 16 ± 12 minutes.

**Table 2:** Left bundle branch area pacing procedural outcomes in patient with right bundle branch block at baseline

Procedural outcomes	
Total number of successful cases	107 (88)
Device indication	
Primary CRT indication	48 (45)
Pacing indication†	48 (45)
RV pacing–induced cardiomyopathy†	5 (5)
AV node ablation†	6 (6)
Procedure duration (min)	97 ± 48
Fluoroscopy duration (min)	16 ± 12
Type of device	
CRT pacemaker	12 (11)
Dual-chamber pacemaker	47 (44)
Single-chamber pacemaker	11 (10)
CRT defibrillator	34 (32)
Dual-chamber defibrillator	3 (3)

Pacing characteristics	Baseline	Follow-up	*P* value
R-wave amplitude (mV)	10 ± 9	13 ± 5	.08
Impedance (Ω)	635 ± 179	478 ± 116	<.01
LBBAP threshold (V at 0.5 ms)	0.8 ± 0.3	0.7 ± 0.3	.38
Stimulus to peak LV activation time (ms)	85 ± 16		
LBBAP pacing burden (%)		95 ± 8	

Values are mean ± SD or n (%).

*P* values <.05 were considered statistically significant.

AV = atrioventricular; CRT = cardiac resynchronization therapy; LBBAP = left bundle branch area pacing; LV = left ventricle; RV = right ventricle.

† Underlying right bundle branch block in sinus bradycardia, AV block, or prior to AV node ablation.

### Left bundle branch capture

Evidence for LBB capture was confirmed in 89 of 107 (83%) patients: LBB potentials were observed in 58 patients (54%) with LBB-V interval of 28 ± 9 ms (Figure 2); nonselective to selective capture in 43 patients (40%); nonselective to LV septal capture in 8 patients (7%); peak LVAT <90 ms in 51 patients (48%); and extrastimulus technique in 18 patients (17%). In 18 (17%) patients, LBB capture could not be confirmed by the described criteria and LV septal pacing was considered to have been achieved in these patients.

Mean capture threshold at implant was 0.8 ± 0.3 V @ 0.5 ms and remained stable at 0.7 ± 0.3 V @ 0.5 ms during a follow-up of 13 ± 8 months (*P* = .38). R-wave amplitude at implant and follow-up were 10 ± 9 mV and 13 ± 5 mV, respectively, while pacing impedance decreased significantly from 635 ± 179 ohms at implant to 478 ± 116 ohms during follow-up (*P* < .01). No immediate procedure-related complications were noted. Lead dislodgement occurred in 1 patient within 24 hours, requiring repositioning, and loss of left bundle / left septal capture was seen in 1 patient at 3-month follow-up.

### Electrocardiographic response

With LBBAP, the QRSd decreased from 156 ± 20 ms at baseline to 150 ± 24 ms (*P* = .01) (Table 3). Complete elimination of RBBB pattern was achieved in 35 patients (33%) (Figure 2). A significant attenuation of RBBB conduction delay pattern (reduction in R′ duration and amplitude) was achieved in 68 (64%) patients (Figure 3A). A normal QRS axis was achieved during LBBAP in 68% of patients compared to 23% at baseline (*P* < .01, Figure 3B), while leftward axis was seen in 28% of patients with LBBAP compared to 63% at baseline (*P* < .01). QRSd reduction by ≥10 ms was observed in 53% of patients. QRSd widening by ≥10 ms was seen in 24% of patients.

**Table 3:** Clinical parameters before and after left bundle branch area pacing

	Baseline	Paced	*P* value
Clinical response: (No HFH + improvement in NYHA class)	53/89 (60%)		
NYHA class	2.5 ± 0.8	1.7 ± 0.8	<.01
Electrocardiographic response:			
QRS duration (mm)	156 ± 20	150 ± 24	.01
Normal axis	25 (23)	73 (68)	<.01
Rightward axis	15 (14)	4 (4)	.01
Leftward axis	67 (63)	30 (28)	<.01
RBBB elimination		35/107 (33%)	
RBBB attenuation		68/107 (64%)	
Echocardiographic response:			
EF improvement by >5%		54/88 (61%)	
Echo super-response		12/48 (25%)	
LVEF	35 ± 9	43 ± 12	<.01
LVEF (≤35% baseline)	27 ± 6	40 ± 11	<.01
LVEF (36%–50% baseline)	42 ± 4	50 ± 8	<.01
LVEDD	54 ± 12	52 ± 12	.17
LVESV	95 ± 46	85 ± 44	.20
LVEDV	136 ± 61	126 ± 54	.31

Values are mean ± SD or n (%).

*P* values <.05 were considered statistically significant.

EF = ejection fraction; HFH = heart failure–related hospitalization; LVEDD = left ventricular end-diastolic diameter; LVEDV = left ventricular end-diastolic volume; LVEF = left ventricular ejection fraction; LVESV = left ventricular end-systolic volume; NYHA = New York Heart Association; RBBB = right bundle branch block.

### Echocardiographic response

Follow-up echocardiographic data were available for 82% of the cohort. Echocardiographic response (≥5% improvement in LVEF) was noted in 61%. In 6 patients (6%), worsening of LV function was observed (≥5% decline in LVEF). Overall LVEF improved significantly from 35% ± 9% at baseline to 43% ± 12% at follow-up (*P* < .01). Among patients with LVEF ≤35%, LVEF improved significantly from 27% ± 6% to 40% ± 11% (*P* < .01). Significant improvement in LVEF was also observed in patients with baseline LVEF of 36%–50% (42% ± 4% to 50% ± 8%, *P* < .01). Super-response was observed in 12 patients (25%). Among 37 patients with follow-up echocardiograms at ≥12 months, LVEF improved from 33% ± 7% at baseline to 44% ± 13% at follow-up (*P* < .01). There were no significant changes in LV end-diastolic dimensions and LV volumes during follow-up (Table 3).

### Clinical outcomes

Clinical response to LBBAP was noted in 60% of patients (defined as improvement in 1 NYHA functional class in the absence of HFH). The NYHA functional class improved from 2.5 ± 0.8 at baseline to 1.7 ± 0.8 on follow-up (*P* < .01). Worsening of functional status (≥1 NYHA class) was observed in 2 patients. During a follow-up of 13 ± 8 months, 9 patients were admitted with HFH (8%), 3 patients developed new-onset atrial fibrillation (3%), and 8 patients (7.5%) died (HF, n = 3; malignancy, n = 2; stroke, n = 1; sepsis, n = 2).

### Subgroup analysis

Among patients undergoing LBBAP for primary CRT indication, normalization of QRS axis was observed in 65% of patients along with nonsignificant reduction in QRS duration from 159 ± 19 ms to 155 ± 24 ms (*P* = .48). NYHA functional class improved from 2.8 ± 0.7 to 2.0 ± 0.9, while LVEF increased from 30% ± 8% to 36% ± 11% (Supplemental Table S1). In patients undergoing LBBAP for primary pacing indication, normalization of QRS axis was observed in 73% of patients, along with significant reduction in QRS duration from 154 ± 21 ms to 145 ± 22 ms (*P* = .04).

### Predictors of response to LBBAP for CRT in RBBB

Univariate analysis of the cohort with successful LBBAP and follow-up echocardiograms (n = 88) showed that female sex, a greater reduction in QRSd during pacing (≥10 ms), and a shorter stimulus to peak LV activation time were predictive of echocardiographic response (Table 4). In a multivariate analysis, female sex and a greater reduction in QRSd during pacing (≥10 ms) remained predictors of echocardiographic response (odds ratio [OR]: 22.87 [95% confidence interval (CI): 2.58–207.73, *P* = .005] and OR: 1.58 [95% CI: 1.21–2.06, *P* = .001], respectively).

**Table 4:** Predictors of echocardiographic response

Echo responder	Univariate				Multivariate 1				Multivariate 2			
Factors	Odds	95% CI lower	95% CI upper	*P* value	Odds	95% CI lower	95% CI upper	*P* value	Odds	95% CI lower	95% CI upper	*P* value
Age	0.977	0.937	1.018	.262								
Female	15.162	1.912	120.24	.01	17.143	1.853	158.57	.012	22.87	2.518	207.73	.005
ICM	0.631	0.265	1.502	.298								
NICM	1.585	0.666	3.773	.298								
LVEDD 5 mm	0.96	0.802	1.149	.655								
LVEF 5%	0.841	0.664	1.065	.151								
Baseline QRS by 10 ms	0.941	0.754	1.174	.588								
Baseline QRS >150 ms	1.018	0.426	2.436	.968								
QRS reduction 10 ms	1.472	1.169	1.855	.001	1.484	1.121	1.964	.006	1.579	1.208	2.064	.001
Stimulus to peak LVAT -5 ms	1.306	1.103	1.546	.002	1.13	0.933	1.37	.211				
RBNA	0.745	0.271	2.05	.569								
RBLA	1.216	0.501	2.949	.666								
RBRA	1.055	0.314	3.542	.932								
RBBB elimination	1.912	0.728	5.023	.189								
RBBB attenuation	0.523	0.199	1.374	.189								
Rescue LBBAP	1.941	0.194	19.46	.573								
Primary CRT indication	0.970	0.410	2.292	.944								

CRT = cardiac resynchronization therapy; ICM = ischemic cardiomyopathy; LBBAP = left bundle branch area pacing; LVAT = left ventricular activation time; LVEDD = left ventricular end-diastolic diameter; LVEF = left ventricular ejection fraction; NICM = nonischemic cardiomyopathy; RBBB = right bundle branch block; RBLA = right bundle branch block with left axis deviation; RBNA = right bundle branch block with normal axis; RBRA = right bundle branch block with right axis deviation.

Univariate analysis identified female sex, reduction of paced QRSd, shorter peak LV activation time, and nonischemic cardiomyopathy as predictors of echocardiographic super-response to LBBAP (Supplemental Table S2). Multivariate analysis revealed that female sex and reduced QRSd with LBBAP predicted echocardiographic super-response (OR: 6.41 [95% CI: 1.12–36.72, *P* = .04] and OR: 1.75 [95% CI: 1.19–2.58, *P* = .005], respectively).

Univariate and multivariate analysis identified female sex and nonischemic cardiomyopathy as predictors of clinical response to LBBAP (OR: 12.69 [95% CI: 1.57–102.75, *P* = .02] and OR: 2.64 [95% CI: 1.03–6.80, *P* = .044], respectively, Table 5). Baseline QRS duration, QRS axis, primary indication for CRT, RBBB elimination, or attenuation during LBBAP did not predict clinical or echocardiographic outcomes.

**Table 5:** Predictors of clinical response

Clinical response	Univariate				Multivariate			
Factors	Odds	95% CI lower	95% CI upper	*P* value	Odds	95% CI lower	95% CI upper	*P* value
Age	0.99	0.954	1.028	.598				
Female	8.028	1.724	37.383	.008	12.692	1.568	102.754	.017
ICM	0.292	0.119	0.714	.007				
NICM	3.43	1.4	8.4	.007	2.643	1.027	6.801	.044
LVEDD 5 mm	0.965	0.809	1.152	.692				
LVEF 5%	1.093	0.872	1.37	.439				
Baseline QRS by 10 ms	0.982	0.793	1.217	.87				
Baseline QRS >150 ms	0.967	0.413	2.265	.938				
QRS reduction 10 ms	1.052	0.885	1.251	.563				
Inverse stimulus to peak 5 ms	0.995	0.864	1.146	.945				
RBNA	1.286	0.454	3.644	.636				
RBLA	1.075	0.44	2.625	.874				
RBRA	0.609	0.179	2.073	.427				
RBBB eliminated	0.68	0.279	1.656	.395				
RBBB attenuated	1.472	0.604	3.585	.395				
Rescue LBBAP	1.02	0.162	6.432	.983				
Primary CRT indication	0.925	0.396	2.162	.857				

Abbreviations as in Table 4.

## Discussion

The main findings of this study of LBBAP in patients with RBBB, reduced LVEF, HF, and indication for CRT or pacing are as follows: (1) LBBAP was associated with significant improvements in LVEF and NYHA functional class, despite only a modest reduction in QRS duration. (2) Female sex and reduction in QRS duration with LBBAP were predictive of echocardiographic response and super-response. (3) LBBAP may be a reasonable alternative strategy for CRT in patients with RBBB and LV dysfunction when QRS narrowing can be achieved at implant.

It is well recognized that BVP results in clinical benefit in patients with HF and cardiac dyssynchrony. This benefit is greatest among patients with LBBB and QRS duration ≥150 ms.^6^ There are conflicting data about the efficacy of BVP-CRT in the non-LBBB HF patients with a QRS duration of 120–150 ms. Meta-analyses and systematic review of data from large randomized controlled trials suggest that patients with RBBB and LV dysfunction do not benefit from conventional BVP-CRT.^7^^,^^8^ Many patients with RBBB may have less apparent disease involving the left-sided conduction system and prolonged atrioventricular (AV) conduction times. In a recent randomized trial of non-LBBB patients with CRT indication (248 patients, 150 with RBBB), BVP resulted in similar response rates (clinical composite score) of 67.2% and 73% in QLV-based LV lead implantation vs standard-of-care anatomic LV lead implantation, respectively. Importantly, both groups showed similar improvement in LVEF of 5.6% and 6% at 12 months, respectively.^22^ Contrary to subgroup analysis of randomized controlled trials and meta-analyses, this prospective randomized study demonstrated significant improvements in both clinical and echocardiographic outcomes in patients with RBBB undergoing conventional BVP-CRT, compared to baseline.

In a previous report, permanent HBP was shown to be feasible and safe in patients with RBBB and CRT indication.^10^ In that study, HBP resulted in correction of underlying RBBB in 78% of patients, while in the remaining patients partial correction via fused RV pacing was achieved. Theoretically, HBP by means of normalizing RV activation while preserving LV activation should be the superior form of electrical resynchronization in patients with RBBB. In our study, HBP was initially attempted in approximately half the population but LBBAP was performed owing to high thresholds, lack of RBBB correction, or inability to map the His bundle. Thus, our study population may be skewed in that 50% of patients failed HBP. In the remaining patients, HBP was not attempted. The true success/failure rates of HBP were not assessed in this study. Only modest reductions in QRS duration were noted with LBBAP (156 ± 20 ms to 150 ± 24 ms) when compared to HBP (158 ± 24 ms to 127 ± 17 ms).^10^ It is likely that many patients with more distal conduction disease were included in our study and proximal conduction disease may slow retrograde conduction back into the right bundle branch, creating more asynchrony. Complete correction of RBBB pattern was observed in 78% of patients in the HBP study compared to only 33% in the current study. Additionally, all the patients in whom LBBAP failed had a QRS duration >150 ms (mean QRS 172 ± 16 ms). Nonetheless, LBBAP resulted in significant clinical and echocardiographic response compared to baseline. Women and those with significant reduction in paced QRS duration had better outcomes.

In LBBAP, LV activation is preserved by means of engaging the LBB conduction system. During selective LBB capture (observed during threshold testing), complete RBBB pattern with significant RV conduction delay remains. The mechanisms for the QRS narrowing observed with LBBAP likely are achieved by nonselective LBB capture, where RV conduction delay is reduced owing to septal myocardial capture. Further reduction in QRS duration and improvement in RV activation (elimination or reduction of RBBB pattern) may be achieved by anodal capture resulting from the ring electrode in good contact with the RV septum.^23^ Anodal capture was used only in 48% of patients owing to thresholds >3 V or no anodal capture in the remaining patients. If pacing configurations of the LBBAP lead can be modified to use both the tip and ring electrodes as cathodes, simultaneous LBB area and RV septal stimulation can be achieved in a much higher proportion of patients. Complete elimination of RBBB pattern was achieved in 33% of patients while significant attenuation of the RBBB delay pattern was seen in the remaining patients. Compared to the abnormal QRS axis at baseline (left 63% and right 14%), LBBAP resulted in a normal QRS axis in 68% of patients. Nonetheless, baseline bifascicular block, normalization of RBBB delay pattern, or normalization of QRS axis did not predict clinical or echocardiographic response to LBBAP in this study. However, reduction in paced QRS duration compared to baseline was an independent predictor of echocardiographic response and super-response to LBBAP. Some of the clinical and echocardiographic improvements in the study population could have occurred owing to medical and other interventions during the course of the study. A randomized controlled study would be necessary to quantify the contribution from LBBAP.

Conventional BVP-CRT in RBBB is associated with the potential risk of LV desynchronization in patients with normal LBB function owing to LV epicardial stimulation associated with reversal of transmural activation. In LBBAP this risk is minimized, as LBB activation of the LV is maintained or improved while RV activation is partially corrected in addition to optimization of AV synchrony. The major advantage of LBBAP over HBP is due to preservation or improvement of LV synchrony at low and stable pacing thresholds, whereas HBP is likely superior to LBBAP in improving RV synchrony. Comparative studies are necessary to determine the most optimal form of conduction system pacing for RBBB.

There may be major benefit from CRT in patients with HF and RBBB and coexistent first-degree AV block. In a sub-study of the MADIT-CRT trial involving 537 patients with a non-LBBB pattern,^24^ in patients with a prolonged PR interval (n = 96), CRT-defibrillator was associated with a 73% reduction in the risk of HF/death (HR = 0.27, *P* < .001) and 81% decrease in the risk of all-cause mortality (HR = 0.19, *P* < .001). In our study, patients with AV conduction disease and need for ventricular pacing and coexistent RBBB had similar clinical and echocardiographic outcomes compared to patients with a primary CRT indication in the absence of AV conduction disease. The clinical impact of the baseline PR interval was not assessed in this study. It is conceivable that AV optimization in addition to bundle branch block correction in patients with prolonged QRS and RBBB may yield additional clinical benefits. This hypothesis was tested in a recently completed randomized clinical trial of HBP (HOPE-HF, NCT02671903); the results are yet to be published.^25^

### Limitations

Despite being a multicenter experience, this was a retrospective study involving nonconsecutive patients with possible selection bias. In addition, the high success rate of LBBAP achieved by experienced operators needs to be reproduced in larger prospective studies. Another limitation of the study is the lack of a direct comparison of conventional BVP-CRT to LBBAP and the lack of a control arm. The results of this retrospective observational study have to be interpreted with caution and the data should be used in a hypothesis-generating fashion to design larger studies.

## Conclusion

Permanent LBBAP was feasible with low capture thresholds in patients with RBBB, LV dysfunction, HF, and an indication for CRT or pacing. LBBAP was associated with significant improvements in LVEF and NYHA functional class. LBBAP may be a reasonable alternative for patients with LV dysfunction and RBBB. Future randomized studies are essential to understand the role of LBBAP in this patient population.
