# Supplementary material for: Left bundle branch area pacing in patients with heart failure and right bundle branch block: Results from International LBBAP Collaborative-Study Group
Source: Heart Rhythm O2. 2022 May 14;3(4):358–67. doi: 10.1016/j.hroo.2022.05.004 (PMC9463705; doi:10.1016/j.hroo.2022.05.004)
Supplement: Supplemental Tables [file mmc1.pptx]

## Slide 1
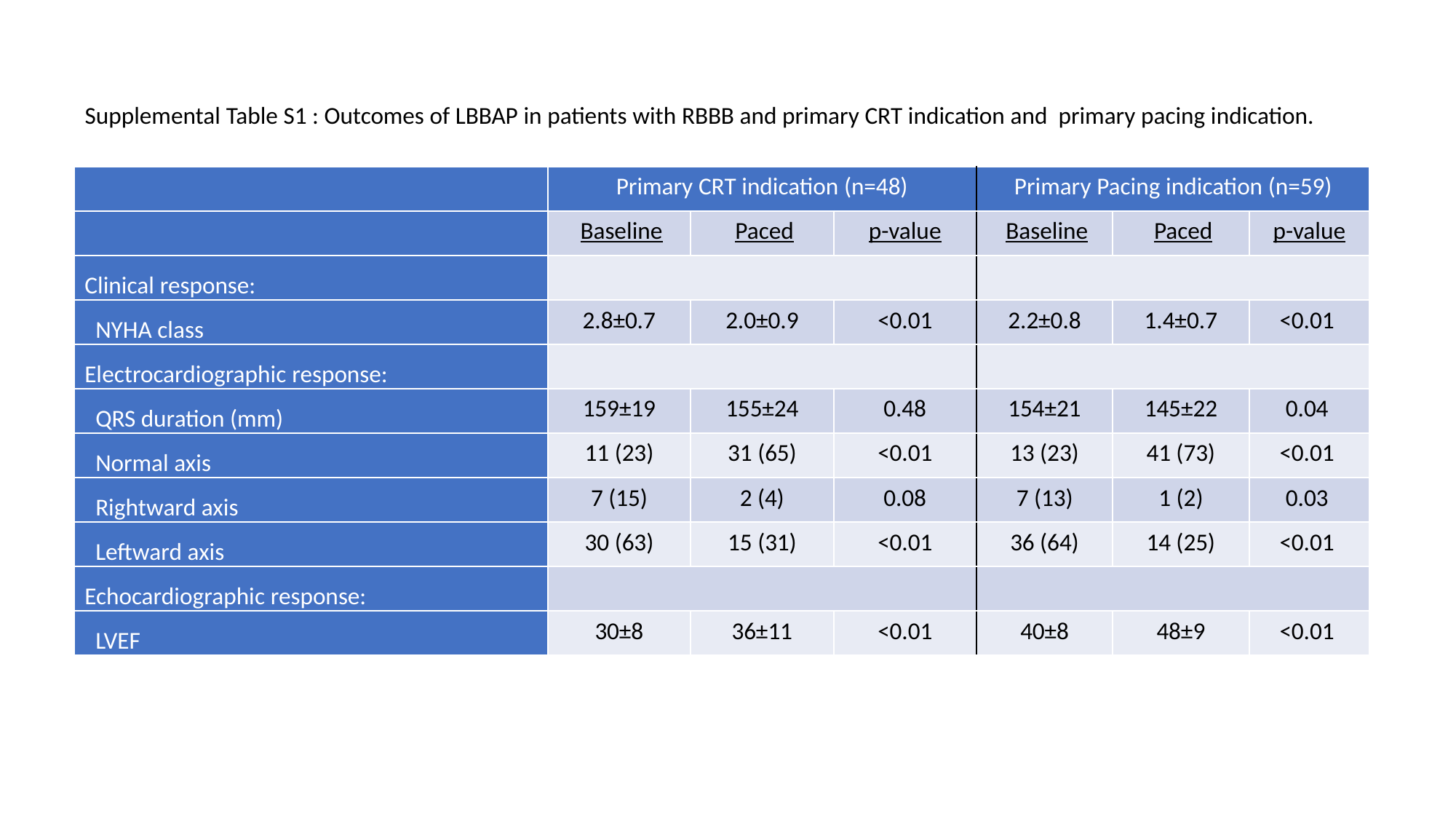

Supplemental Table S1 : Outcomes of LBBAP in patients with RBBB and primary CRT indication and primary pacing indication.
| | Primary CRT indication (n=48) | | | Primary Pacing indication (n=59) | | |
| --- | --- | --- | --- | --- | --- | --- |
| | Baseline | Paced | p-value | Baseline | Paced | p-value |
| Clinical response: | | | | | | |
| NYHA class | 2.8±0.7 | 2.0±0.9 | <0.01 | 2.2±0.8 | 1.4±0.7 | <0.01 |
| Electrocardiographic response: | | | | | | |
| QRS duration (mm) | 159±19 | 155±24 | 0.48 | 154±21 | 145±22 | 0.04 |
| Normal axis | 11 (23) | 31 (65) | <0.01 | 13 (23) | 41 (73) | <0.01 |
| Rightward axis | 7 (15) | 2 (4) | 0.08 | 7 (13) | 1 (2) | 0.03 |
| Leftward axis | 30 (63) | 15 (31) | <0.01 | 36 (64) | 14 (25) | <0.01 |
| Echocardiographic response: | | | | | | |
| LVEF | 30±8 | 36±11 | <0.01 | 40±8 | 48±9 | <0.01 |

## Slide 2
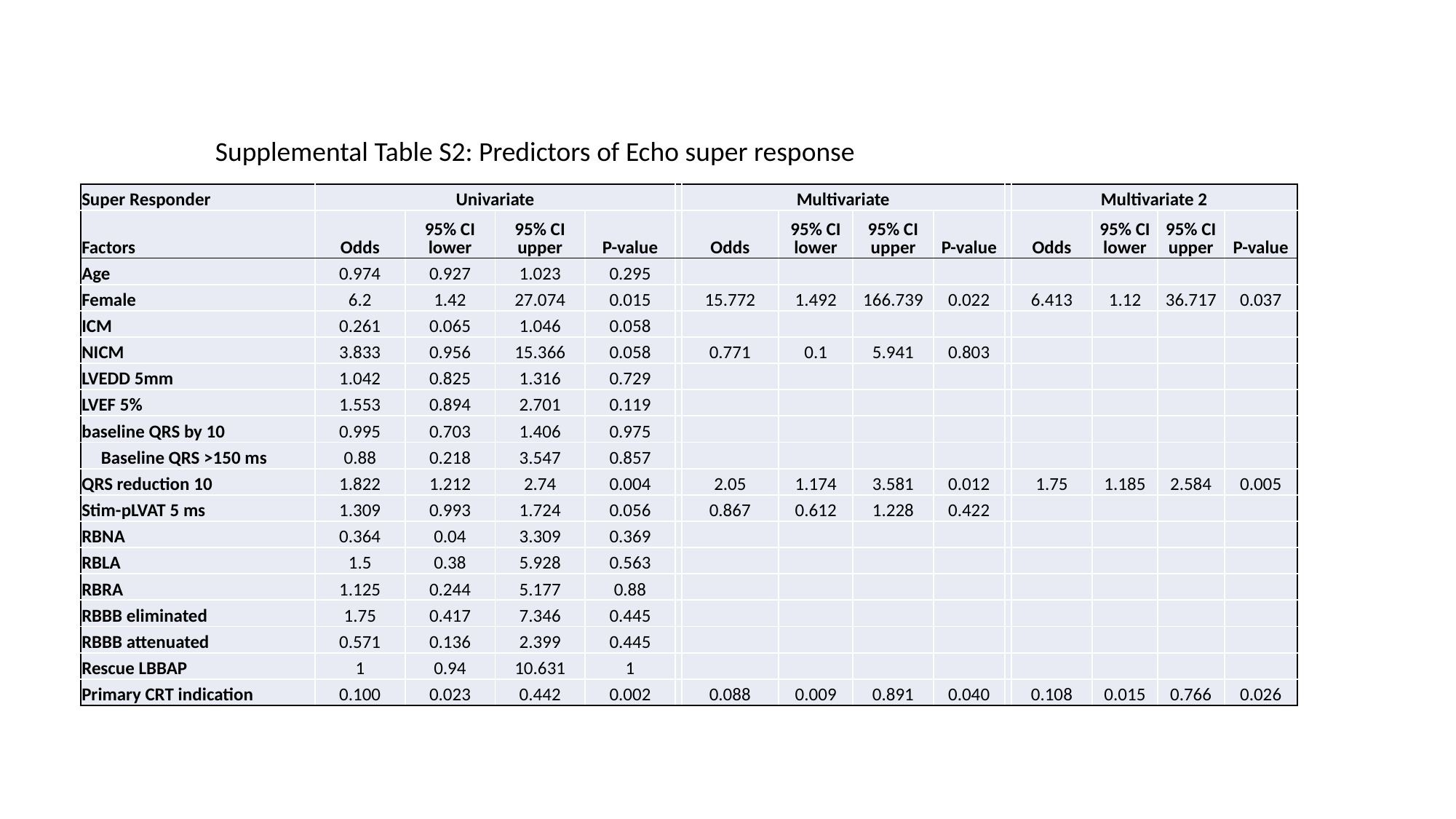

Supplemental Table S2: Predictors of Echo super response
| Super Responder | Univariate | | | | | Multivariate | | | | | Multivariate 2 | | | |
| --- | --- | --- | --- | --- | --- | --- | --- | --- | --- | --- | --- | --- | --- | --- |
| Factors | Odds | 95% CI lower | 95% CI upper | P-value | | Odds | 95% CI lower | 95% CI upper | P-value | | Odds | 95% CI lower | 95% CI upper | P-value |
| Age | 0.974 | 0.927 | 1.023 | 0.295 | | | | | | | | | | |
| Female | 6.2 | 1.42 | 27.074 | 0.015 | | 15.772 | 1.492 | 166.739 | 0.022 | | 6.413 | 1.12 | 36.717 | 0.037 |
| ICM | 0.261 | 0.065 | 1.046 | 0.058 | | | | | | | | | | |
| NICM | 3.833 | 0.956 | 15.366 | 0.058 | | 0.771 | 0.1 | 5.941 | 0.803 | | | | | |
| LVEDD 5mm | 1.042 | 0.825 | 1.316 | 0.729 | | | | | | | | | | |
| LVEF 5% | 1.553 | 0.894 | 2.701 | 0.119 | | | | | | | | | | |
| baseline QRS by 10 | 0.995 | 0.703 | 1.406 | 0.975 | | | | | | | | | | |
| Baseline QRS >150 ms | 0.88 | 0.218 | 3.547 | 0.857 | | | | | | | | | | |
| QRS reduction 10 | 1.822 | 1.212 | 2.74 | 0.004 | | 2.05 | 1.174 | 3.581 | 0.012 | | 1.75 | 1.185 | 2.584 | 0.005 |
| Stim-pLVAT 5 ms | 1.309 | 0.993 | 1.724 | 0.056 | | 0.867 | 0.612 | 1.228 | 0.422 | | | | | |
| RBNA | 0.364 | 0.04 | 3.309 | 0.369 | | | | | | | | | | |
| RBLA | 1.5 | 0.38 | 5.928 | 0.563 | | | | | | | | | | |
| RBRA | 1.125 | 0.244 | 5.177 | 0.88 | | | | | | | | | | |
| RBBB eliminated | 1.75 | 0.417 | 7.346 | 0.445 | | | | | | | | | | |
| RBBB attenuated | 0.571 | 0.136 | 2.399 | 0.445 | | | | | | | | | | |
| Rescue LBBAP | 1 | 0.94 | 10.631 | 1 | | | | | | | | | | |
| Primary CRT indication | 0.100 | 0.023 | 0.442 | 0.002 | | 0.088 | 0.009 | 0.891 | 0.040 | | 0.108 | 0.015 | 0.766 | 0.026 |
